# Characterization of a Novel Tomato R2R3-MYB Transcription Factor Gene, *SlMYB306-like*, Conferring Salt Tolerance in *Arabidopsis*

**DOI:** 10.3390/biology14111566

**Published:** 2025-11-07

**Authors:** Guohua Cai, Tianqi Chen, Wenjing Wang, Luming Wang, Zhaowei Yin, Jingrui He, Jiadong Gao, Guodong Wang

**Affiliations:** School of Life Sciences, Jining Medical University, Rizhao 276800, China; 13375545350@163.com (T.C.); wj371723@163.com (W.W.); 19819520644@163.com (L.W.); 13370640519@163.com (Z.Y.); 13707681873@163.com (J.H.); 15054414416@163.com (J.G.)

**Keywords:** tomato, SlMYB306-like, transcription factor, *Arabidopsis*, salt stress

## Abstract

**Simple Summary:**

Soil salinization is a serious global issue that limits plant growth, reduces crop yields, and threatens agricultural productivity. As a result, plants have developed sophisticated adaptive strategies to improve salt tolerance for sustainable agricultural development. The MYB transcription factor family is one of the largest in plants and is integral to the regulation of physiological and biochemical processes under abiotic and biotic stresses. This study presents the first isolation and characterization of a new tomato R2R3-MYB factor, SlMYB306-like, which is shown to enhance salt tolerance by modulating both plant growth and physiological parameters such as photosynthetic efficiency, osmotic adjustment substance levels, and antioxidant enzyme activities. Mechanistically, SlMYB306-like mediated salt tolerance by upregulating key genes involved in ROS scavenging and salt stress responses. Overall, the results reveal SlMYB306-like as a critical regulator of salt tolerance, providing a valuable genetic resource for developing salt-tolerant crops and boosting yields in high-salinity soils, in turn supporting more stable food production.

**Abstract:**

Soil salinization significantly limits plant growth and agricultural productivity, with MYB transcription factors playing crucial roles in mediating plant responses to salt stress. In this study, a novel R2R3-MYB transcription factor gene, *SlMYB306-like*, was isolated from tomato. Phylogenetic comparison indicated that SlMYB306-like shared the highest sequence homology with potato StMYB306-like. Subcellular localization assays demonstrated nuclear localization of SlMYB306-like protein, while yeast transactivation assays confirmed its function as a transcriptional activator. Expression profiling showed that *SlMYB306-like* was inducible by NaCl and abscisic acid (ABA) treatments. In addition, functional characterization via the overexpression of *SlMYB306-like* in *Arabidopsis thaliana* revealed enhanced salt tolerance, evidenced by an increased maximum quantum efficiency of photosystem II (Fv/Fm) and proline levels alongside decreased accumulation of reactive oxygen species (ROS) and malondialdehyde (MDA) content under salt stress conditions. Furthermore, the overexpression of *SlMYB306-like* upregulated the expression of several stress-responsive genes, including *AtSOD1*, *AtCAT1*, *AtEGY3*, *AtP5CS2*, and *AtRD29A*. Collectively, these findings suggest that SlMYB306-like enhances salt tolerance by modulating ROS scavenging, osmotic adjustment, and ABA signaling pathways, thereby representing a promising candidate gene for the development of salt-tolerant crops.

## 1. Introduction

Soil salinization represents a significant global challenge. Elevated concentrations of salt ions, predominantly sodium ions (Na^+^), in the soil can induce salt damage or salt stress in plants, thereby impairing their normal growth and development. This phenomenon leads to substantial agricultural losses and severe degradation of plant ecosystems [1]. Under salt stress conditions, plants commonly exhibit symptoms such as reduced germination rates, stunted growth, diminished plant height, and chlorosis of leaves [2]. Initially, salt stress induces osmotic stress in plants, which hampers their capacity to absorb water and nutrients from the soil, consequently affecting their growth and developmental processes [3]. Furthermore, salt ions absorbed by plants interfere with the transpiration stream, resulting in ionic toxicity and the accumulation of ROS. These effects damage membrane lipids through peroxidation and disrupt ionic homeostasis, thereby inhibiting photosynthesis and diminishing the growth and developmental potential of plants [4]. Hence, enhancing plant stress resistance is essential for optimizing plant productivity and ensuring sustainable agricultural development [5].

When plants encounter environmental stressors, extracellular signals are transmitted into the cells via signal transduction pathways. These signals interact with proteins associated with the plant defense system, leading to the activation or repression of downstream defense-related genes, thereby enabling plants to respond to adverse environmental conditions [6]. Among the molecular components involved, transcription factors play a critical role in regulating plant responses to environmental stimuli. Notably, several transcription factor families, including MYB, NAC, WRKY, AP2/ERF, and bZIP, have been extensively characterized. Within this diverse group, the MYB transcription factor family is one of the largest in plants and is integral to the regulation of responses to both abiotic and biotic stresses, participating in extensive physiological and biochemical plant processes [7].

The first identified plant MYB gene, *COLORED1* (*C1*), was isolated from *Zea mays* in 1987 and is involved in the complex regulation of anthocyanin biosynthesis through modulation of the structural genes *Bz1* and *A1* [8]. The MYB transcription factor family is defined by the presence of a highly conserved MYB domain located at the N-terminus that facilitates DNA binding [9]. This domain typically comprises one to four imperfect repeats (designated R), each approximately 52 amino acids in length, folding into three α-helices that determine the specificity of the transcription factor binding to target DNA elements [9,10]. Although the C-terminal region is less conserved than the N-terminal domain, it contains a transcriptional activation domain responsible for maintaining transcriptional activity and mediating interactions with other transcription factors or DNA sequences. This structural organization underlies the diverse regulatory functions attributed to the MYB gene family [11]. According to the number of adjacent repeats within the MYB domain, MYB family members are divided into four subgroups: R1/R2-MYB (containing a single or partial repeat), R2R3-MYB (two repeats), R1R2R3-MYB (three repeats), and 4R-MYB (four repeats). Among these, R2R3-MYB transcription factors constitute the predominant subgroup within the MYB family [12].

With ongoing advancements in biological research, investigations into the MYB transcription factor family have proliferated, revealing that numerous MYB proteins play important roles in plant organogenesis, primary and secondary metabolic pathways, and cell fate and identity determination, as well as responses to biotic and abiotic stresses. For instance, in root hair cells of *Arabidopsis*, the 2R-MYB transcription factor WER can suppress root hair cell differentiation, whereas the 3R-MYB factor CPC can promote this differentiation [13]. During plant growth and development, *Arabidopsis* AtMYB20, AtMYB42, and AtMYB43 regulate secondary cell wall formation by activating genes involved in lignin biosynthesis [14]. In tomato, SlMYB117 influences leaf development and modulates levels of γ-aminobutyric acid, carotenoids, and folic acid, thereby affecting fruit ripening [15]. Additionally, the SlMYB15 transcription factor enhances cold tolerance through heterodimerization with the transcription factor HY5 [16]. In apple, MdMYB88 and MdMYB124 can contribute to drought stress responses by modulating root system architecture [17], whereas in wheat, the MYB transcription factor TaRIM1 participates in pathogen resistance by positively regulating defense-related genes such as *PR10* and *PR17c* [18].

Tomato (*Solanum lycopersicum* L.) is a globally significant horticultural crop and serves as a widely utilized model organism for genetic transformation studies across various disciplines. Environmental stressors such as high salinity, drought, low temperature, and pathogen infection substantially impair tomato growth, development, and yield. To date, 127 R2R3-MYB transcription factors have been identified in tomato, but only a few of them have been functionally characterized; for example, SlMYB15 is induced by low temperature and SlMYB117 affects leaf development [15,16,19]. In the current study, a novel R2R3-MYB transcription factor gene that was induced by salt stress, *SlMYB306-like*, was successfully isolated. Additionally, its functional role and regulatory mechanism under salt stress conditions were thoroughly investigated. This research not only advances the understanding of plant salt tolerance mechanisms but also provides a theoretical foundation for crop improvement strategies, including breeding and genetic engineering aimed at enhancing salt tolerance.

## 2. Materials and Methods

### 2.1. Plant Materials, Growth Conditions, and Treatment

Wild-type (WT) *Arabidopsis thaliana* (Columbia-0) and T_3_ generation transgenic *Arabidopsis* plants, generated through genetic transformation conducted in our laboratory, were cultivated in quartz sand at 22 °C under a 14 h light/10 h dark photoperiod, with a photon flux density of 200 µmol m^−2^ s^−1^. The WT tomato cultivar, maintained in our laboratory, was grown in quartz sand at 25 °C under a 16 h light/8 h dark photoperiod, also with a photon flux density of 200 µmol m^−2^ s^−1^. All plant materials were irrigated weekly with Hoagland’s nutrient solution.

To examine the expression pattern of *SlMYB306-like* in response to salt stress, roots from six-week-old WT tomato plants were treated with Hoagland’s nutrient solution supplemented with 250 mM NaCl for 0, 1, 2, 3, 4, 5, 6, and 7 d, while control plants received only Hoagland’s solution. For ABA treatment, leaves were sprayed with 100 µM ABA, with control plants sprayed with water. Salt tolerance assays were performed by applying 200 mM NaCl solution to three-week-old WT and T_3_ transgenic *Arabidopsis* plants for a duration of ten days.

### 2.2. Isolation and Bioinformatic Characterization of SlMYB306-like

The open reading frame (ORF) sequence of *SlMYB306-like* was amplified from cDNA synthesized from leaves of WT tomato plants using PCR with specific primers (Table S1). The resulting PCR products were subsequently connected into the pMD19-T vector (TaKaRa, Beijing, China) for sequencing. DNAMAN 10.0 software (Lynnon Biosoft, San Ramon, CA, USA) was employed for multiple sequence alignments, and MEGA 7.0 software (Sudhir Kumar, Temple University, Philadelphia, PA, USA) was used to perform phylogenetic analysis of the SlMYB306-like protein. SMART program (version 9.0. http://smart.embl-heidelberg.de/ (accessed on 26 September 2024) was used to perform domain analysis, and SOPMA program (https://npsa-prabi.ibcp.fr/cgi-bin/npsa_automat.pl?page=/NPSA/npsa_sopma.html (accessed on 20 September 2024) was used to predict the protein’s secondary structure. Moreover, the MYB protein amino acid sequences of various plant species were retrieved from GenBank to facilitate comparative analyses.

### 2.3. Subcellular Localization of SlMYB306-like

The complete coding region of *SlMYB306-like* was amplified using cloning-specific primers (Table S1). Following amplification, the DNA fragments were cloned into *BamH*I and *Xba*I sites of the pCAMBIA1300-EGFP vector to generate a *35S*::*SlMYB306-like*-EGFP fusion construct. Both the EGFP control plasmid and SlMYB306-like-EGFP recombinant plasmid were introduced into *Agrobacterium tumefaciens* strain *LBA4404*. Tobacco leaf epidermal cells were then individually infiltrated with these *Agrobacterium* strains, and fluorescence signals were detected using a Leica TCS SP8 laser confocal microscope (Leica, Wetzlar, Germany).

### 2.4. Yeast Transactivation Assay of SlMYB306-like

The transcriptional activation potential of SlMYB306-like was evaluated utilizing a yeast-based system, and its complete coding sequence was amplified by employing primers containing *Nde*I and *BamH*I restriction sites and subsequently cloned into the pGBKT7 vector (Clontech) to generate the expression construct pGBKT7-*SlMYB306-like*. The fusion construct pBD-*SlMYB306-like*, empty pGBKT7 vector (negative control), and pGAL4 (positive control) were each transformed into the yeast strain *AH109*. The transformed yeast cells were incubated on a tryptophan-deficient SD medium (SD/-Trp) at 30 °C for 48 h. Following this, the yeast strains were transferred onto a selective SD medium deficient in both tryptophan and histidine (SD/-Trp/-His) and incubated under the same conditions for an additional 48 h to assess transcriptional activation.

### 2.5. Molecular Cloning of the SlMYB306-like Promoter and GUS Staining Analysis

The promoter region of *SlMYB306-like* was isolated using the cetyltrimethylammonium bromide (CTAB) extraction method, with genomic DNA extracted from leaves of WT tomato plants serving as the template. For histochemical analysis, GUS staining was performed according to established protocols as described in the prior literature [20].

### 2.6. Transformation and Generation of Transgenic Lines

The coding sequence of *SlMYB306-like* was constructed into the pCAMBIA1302 binary vector, and the resulting recombinant plasmid was then introduced into WT Arabidopsis plants using *Agrobacterium*-mediated floral dip methods. Transgenic seedlings were screened on the 1/2 MS hygromycin-containing (25 µg/mL) medium, and the positive transgenic lines were verified by PCR using the 35S promoter forward and *SlMYB306-like* reverse primers (Table S1). Subsequently, ten independent T_3_ homozygous lines over-expressing *SlMYB306-like* were established. To ensure experimental consistency, all seeds used in the assays were harvested at the same developmental stage and stored under identical conditions.

### 2.7. Salt Stress Treatment

In the salt stress assay, surface-sterilized seeds from the T_3_ generation and WT progeny were sown on a 1/2 MS medium supplemented with NaCl at concentrations of 0, 100, 125, and 150 mM. Following a 2-day vernalization period at 4 °C in darkness, the plates were transferred to a growth chamber and germination rates were recorded daily. To evaluate root growth post-germination, seeds were initially germinated on a 1/2 MS medium for 3 days, and seedlings exhibiting radicle emergence were then transferred to a 1/2 MS medium containing 0, 100, or 150 mM NaCl for an additional 5 days. Subsequent assessments included root elongation and fresh weight measurements. To evaluate salt tolerance in mature plants, 3-week-old WT and T_3_ transgenic plants were continuously treated with 200 mM NaCl for 10 days, after which phenotypic assessments were performed. In the germination experiment, seeds of each genotype were subjected to varying salt concentration treatments, with 49 seeds placed in each Petri dish and three Petri dishes allocated per treatment, resulting in a total number of 147 seeds per genotype in each experimental condition. In the root length assessment, five seedlings of each genotype were cultivated in each of three Petri dishes under different salt concentration treatments, resulting in a total of 15 seedlings per genotype for each experiment. For the phenotypic observation during seedling establishment, three plants per genotype were treated, with three replicates conducted for each experiment, yielding a total of nine established seedlings per genotype in each experimental trial. Chlorophyll content was quantified according to previously established protocols [21], and all experiments were conducted in triplicate.

### 2.8. Analysis of Chlorophyll Fluorescence Characteristics

A FluorCam closed fluorescence imaging system (PSI, Norfolk, VA, USA) was used to perform chlorophyll fluorescence imaging following established methodologies [22]. Additionally, a Handy PEA instrument (Hansatech Instruments, Norfolk, UK) was used to measure chlorophyll fluorescence parameters as described in prior studies [23]. The maximum photochemical efficiency of PSII, denoted as Fv/Fm, was calculated using the following formula: Fv/Fm = (Fm − Fo)/Fm.

### 2.9. Histochemical Analysis

For the detection of hydrogen peroxide (H_2_O_2_) and superoxide anion radicals (O_2_^•−^), leaves were incubated in diaminobenzidine (DAB) staining solution (0.1 mg/mL DAB dissolved in 50 mM Tris-acetic acid buffer, pH 5.0) and nitroblue tetrazolium (NBT) staining solution (0.1 mg/mL NBT dissolved in 25 mM phosphate buffer, pH 7.6), respectively. Incubation was performed overnight at room temperature in the dark. Subsequently, leaves were decolorized by boiling for 10 min in a bleaching solution composed of ethanol, acetic acid, and glycerol in a 4:1:1 ratio, followed by photographic documentation. Trypan blue staining was conducted according to previously described protocols [24]. For ROS staining, 4-week-old WT and T_3_ transgenic *Arabidopsis* plants were treated with 200 mM NaCl for 2 days, whereas trypan blue staining was performed after 5 days of the same treatment.

### 2.10. Physiological Indicator Determination

Four-week-old WT and T_3_ transgenic *Arabidopsis* plants were treated with 200 mM NaCl for 2 days prior to physiological analyses. H_2_O_2_ and O_2_^•−^ levels in leaf tissues were quantified following established methods [21], and malondialdehyde (MDA) content and relative electrolyte conductivity (REC) were also determined as described previously [21]. Proline content was measured using a modified acidic ninhydrin assay [25] and soluble sugar content was assessed via the Komas Brilliant Blue method utilizing a commercial plant soluble sugar assay kit (Nanjing Jiancheng, Nanjing, China). Activities of SOD (EC 1.15.1.1) and CAT (EC 1.11.1.6) were determined according to protocols outlined in prior research [26].

### 2.11. Fluorescence Quantitative PCR Analysis

Total RNA was extracted from leaf samples using the MolPure^®^ Plant RNA Kit (Yeasen, Shanghai, China). Complementary DNA (cDNA) synthesis was performed using the PrimeScript RT Reagent Kit (TaKaRa, Kusatsu, Japan), and qPCR was conducted employing the SYBR Green Premix Ex Taq Kit (TaKaRa, Kusatsu, Japan) on a StepOnePlus Real-Time PCR System (Thermo Fisher, Waltham, MA, USA). The reference genes utilized were elongation factor 1-alpha (*EF-1α*; GenBank Accession No.: LOC544055) and *Arabidopsis thaliana* ubiquitin gene *AtUbiquitin* (Gene ID: At4g05320). The qPCR cycling conditions comprised an initial denaturation at 95 °C for 30 s followed by 40 cycles of denaturation at 95 °C for 5 s, then annealing/extension at 55 °C (primer-specific melting temperature) for 30 s, concluding with a melting curve analysis from 65 °C to 95 °C with continuous fluorescence acquisition. Primer sequences are provided in Table S1. For the qPCR assay, four-week-old WT and T_3_ transgenic *Arabidopsis* plants were treated with a 200 mM NaCl solution for 2 days.

### 2.12. Statistical Analysis

Statistical analyses were performed using OriginPro 8.0 (OriginLab, Northampton, MA, USA) and SPSS 18.0 (Chicago, IL, USA). Data are presented as means ± standard deviations derived from three or more independent replicates. Student’s *t*-test was employed to conduct the statistical analysis, where statistical significance was denoted by * for *p* < 0.05 and ** for *p* < 0.01.

## 3. Results

### 3.1. Isolation and Bioinformatics Characterization of the SlMYB306-like Gene

Sequence analysis revealed that the ORF of the *SlMYB306-like* gene comprises 879 base pairs, encoding a polypeptide of 292 amino acids, and computational analysis using the ExPASy tool (version 3.0) predicted the molecular weight of the encoded protein to be 33.06 kDa, with a theoretical isoelectric point (pI) of 6.25. Among the twenty amino acids, serine (11.6%), leucine (8.2%), threonine (7.9%), and lysine (7.2%) were the most abundant residues. The instability index was calculated as 50.43, the aliphatic index as 60.21, and the grand average of hydropathicity (GRAVY) as approximately −0.815, indicating that the protein is hydrophilic and predicted to be unstable.

**Figure 1:**
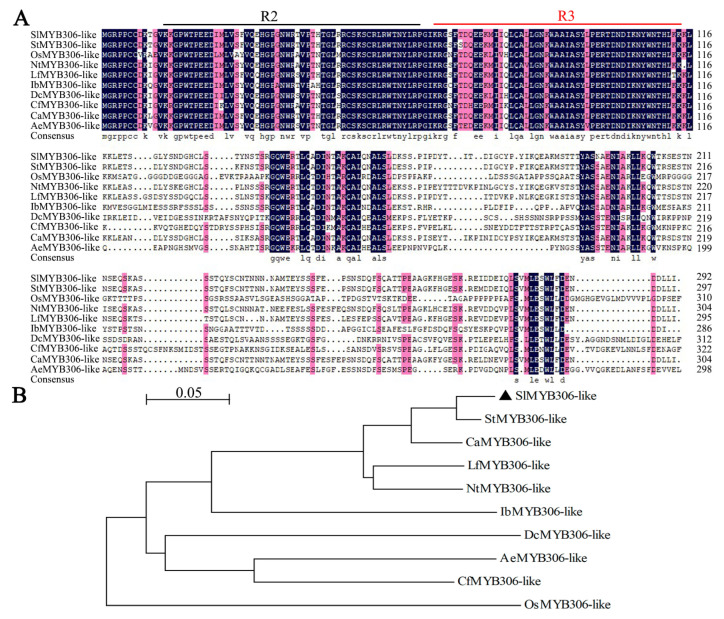
Homologous sequence alignment and phylogenetic analysis of MYB proteins. (**A**) Multiple sequence alignment of R2R3-type MYB transcription factors derived from various species. Conserved R2R3 domains are indicated by underlining. (**B**) Phylogenetic tree depicting MYB306-like proteins across different species. The tomato MYB306-like protein is denoted by a triangle. The unrooted phylogenetic tree was constructed using the neighbor-joining method implemented in MEGA software (version 7.0). Accession numbers are as follows: SlMYB306-like (*Solanum lycopersicum*, XP_004232220); StMYB306-like (*Solanum tuberosum*, XP_006338450); AeMYB306-like (*Actinidia eriantha*, XP_057491139); CaMYB306-like (*Capsicum annuum*, XP_016562275); CfMYB306-like (*Cornus florida*, XP_059670329); DcMYB306-like (*Dendrobium catenatum*, XP_020674883); IbMYB306-like (*Ipomoea batatas*, GMC90254); LfMYB306-like (*Lycium ferocissimum*, XP_059293978); NtMYB306-like (*Nicotiana tabacum*, XP_016451337); OsMYB306-like (*Oryza sativa*, XP_015648957).

A BLAST (version 2.16.0) search of the SlMYB306-like protein sequence against the NCBI database identified ten homologous proteins from diverse species. These homologs, including StMYB306-like, CaMYB306-like, NtMYB306-like, LfMYB306-like, OsMYB306-like, IbMYB306-like, DcMYB306-like, AeMYB306-like, and CfMYB306-like, alongside SlMYB306-like, were subjected to multiple sequence alignment and phylogenetic analysis (Figure 1A,B). The alignment revealed that SlMYB306-like, consistent with other members of the R2R3-MYB subfamily, contains a conserved R2R3 domain at the N-terminus (Figure 1A). In contrast, the C-terminal regions exhibited greater sequence divergence among the MYB306 homologs. Phylogenetic reconstruction demonstrated that SlMYB306-like clusters closely with StMYB306-like, sharing the highest sequence similarity (97.53%) and suggesting conserved biological functions between these two proteins (Figure 1B).

### 3.2. Structural Prediction of the SlMYB306-like Protein

**Figure 2:**
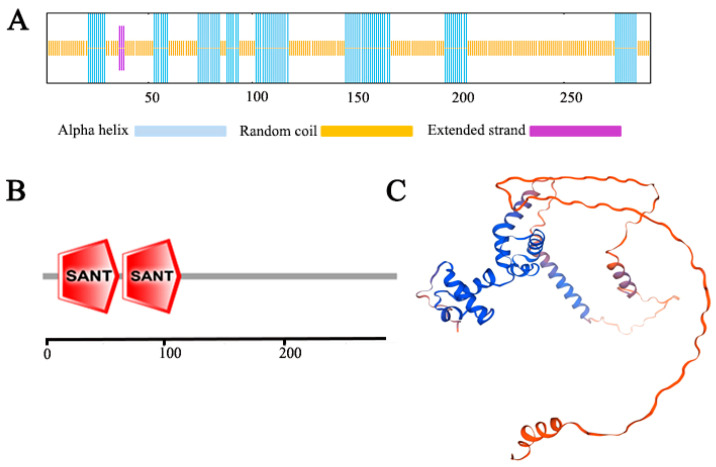
Structural characterization of the SlMYB306-like protein. (**A**) Predicted secondary structure. (**B**) Domain architecture. (**C**) Predicted tertiary structure of the SlMYB306-like protein.

Domain analysis using the SMART program identified two SANT domains within the SlMYB306-like protein, positioned at residues 13–63 and 66–114, consistent with the canonical features of the R2R3-MYB subfamily (Figure 2A). Secondary structure prediction via SOPMA indicated that, out of the 292 amino acids, 190 residues (65.07%) form irregular coils, 99 residues (33.90%) constitute alpha helices, and 3 residues (1.03%) adopt extended strand conformations (Figure 2B). Thus, the secondary structure predominantly comprises random coils and alpha helices, with a minor proportion of extended strands. Tertiary structure modeling using Swiss-Model corroborated these findings, revealing a protein architecture mainly composed of irregular coils and alpha helices (Figure 2C). Collectively, these structural analyses support the classification of SlMYB306-like as a member of the R2R3-MYB transcription factor subgroup.

### 3.3. Subcellular Localization and Transcriptional Activation Activity of SlMYB306-like

**Figure 3:**
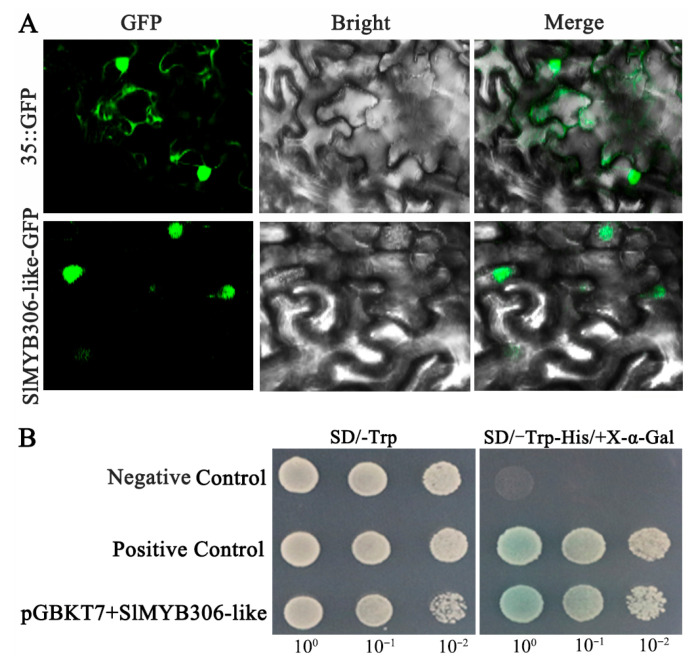
Subcellular localization and transcriptional activation analysis of the SlMYB306-like protein. (**A**) Transient expression of 35S-EGFP (upper panel) and SlMYB306-like-EGFP fusion protein (lower panel) in tobacco epidermal cells. (**B**) Yeast strain AH109 expressing pGAL4 (positive control), pGBKT7 (negative control), and pGBKT7-SlMYB306-like (fusion construct), respectively.

The subcellular localization of the tomato SlMYB306-like protein was predicted using ProtComp 9.0 (http://www.softberry.com/berry.phtml?topic=protcomppl&group=programs&subgroup=proloc (accessed on 10 October 2024), which indicated nuclear localization (Table S2). To experimentally confirm this prediction, the ORF of *SlMYB306-like*, excluding the stop codon, was fused to the enhanced green fluorescent protein (EGFP) and transiently expressed in Nicotiana tabacum leaves. Confocal microscopy revealed that the fluorescence signal of the SlMYB306-like::EGFP fusion protein was specifically localized within the nuclei, whereas the control EGFP fluorescence was distributed throughout the entire cell (Figure 3A). These findings confirm that SlMYB306-like is localized in the nucleus, consistent with its putative function as a transcription factor.

In addition to nuclear localization, transcription factors are characterized by their ability to activate transcription. To assess the transcriptional activation potential of SlMYB306-like, yeast transactivation assays were performed. Constructs encoding the pGBKT7-SlMYB306-like fusion protein, the negative control pGBKT7 vector, and the positive control pGAL4 were individually transformed into the yeast strain AH109. All transformed yeast cells exhibited robust growth on the SD/-Trp medium. Notably, yeast cells harboring pGBKT7-*SlMYB306-like* and the positive control pGAL4 were capable of growth on the SD/-Trp/-His/X-α-Gal medium, whereas cells containing the negative control pGBKT7 failed to grow under these conditions (Figure 3B). These results demonstrate that SlMYB306-like possesses transcriptional activation activity, supporting its role as a transcription factor.

### 3.4. Expression Pattern of SlMYB306-like

**Figure 4:**
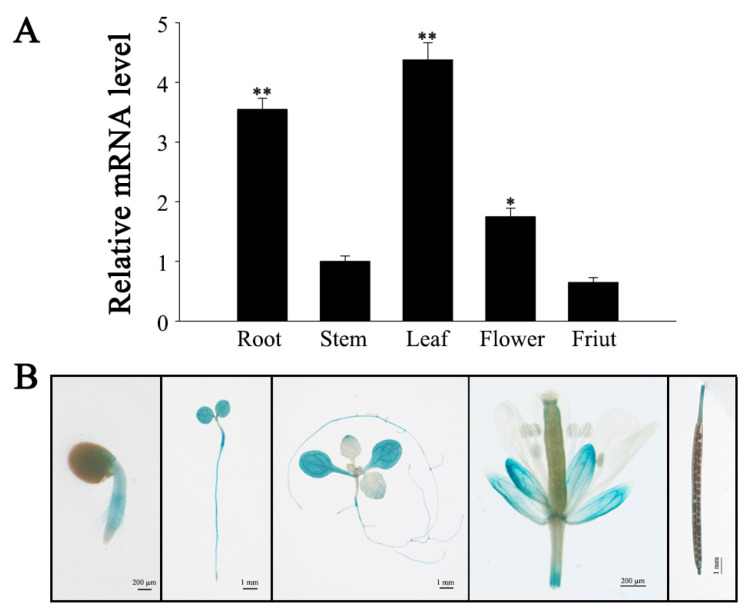
Tissue-specific expression analysis of *SlMYB306-like*. (**A**) qPCR analysis of *SlMYB306-like* transcript levels across various tomato tissues. (**B**) GUS staining of *SlMYB306-like* promoter-driven GUS expression in different tissues of transgenic *Arabidopsis thaliana*. Statistical significance is indicated by * *p* < 0.05 and ** *p* < 0.01.

**Figure 5:**
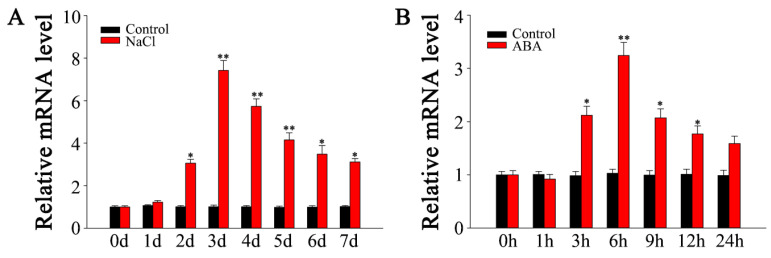
Induction of SlMYB306-like expression by NaCl and ABA treatments. (**A**) Expression response to NaCl. (**B**) Expression response to ABA. Statistical significance is indicated by * *p* < 0.05 and ** *p* < 0.01.

The expression profile of *SlMYB306-like* across various tomato tissues was examined via quantitative PCR (qPCR). As illustrated in Figure 4A, *SlMYB306-like* expression was highest in leaves, followed by roots. To further validate these findings, β-glucuronidase (GUS) staining was conducted to visualize *SlMYB306-like* expression in different tissues (Figure 4B), consistent with the qPCR data. Upon exposure to 250 mM NaCl, *SlMYB306-like* transcript levels peaked at day three post-treatment before declining (Figure 5A). Furthermore, treatment with 100 µM ABA induced *SlMYB306-like* expression, reaching a maximum at 6 h post-treatment (Figure 5B). Collectively, these data indicate that *SlMYB306-like* expression is markedly upregulated in response to salt stress.

### 3.5. Overexpression of SlMYB306-like Confers Enhanced Salt Tolerance in Arabidopsis

To elucidate the functional role of SlMYB306-like in *Arabidopsis* under NaCl stress conditions, transgenic *Arabidopsis* lines overexpressing *SlMYB306-like* were generated using genetic transformation techniques. A total of ten hygromycin-resistant transgenic lines were successfully established and their transgene expression was confirmed via qPCR analysis. Homozygous T_2_ plants exhibiting no segregation on selective media were selected to produce T_3_ homozygous lines for subsequent experimental analyses. Among these, three transgenic lines, designated OE1, OE4, and OE5, were chosen for further study based on their differential relative expression levels as determined by qPCR (Figure S1).

**Figure 6:**
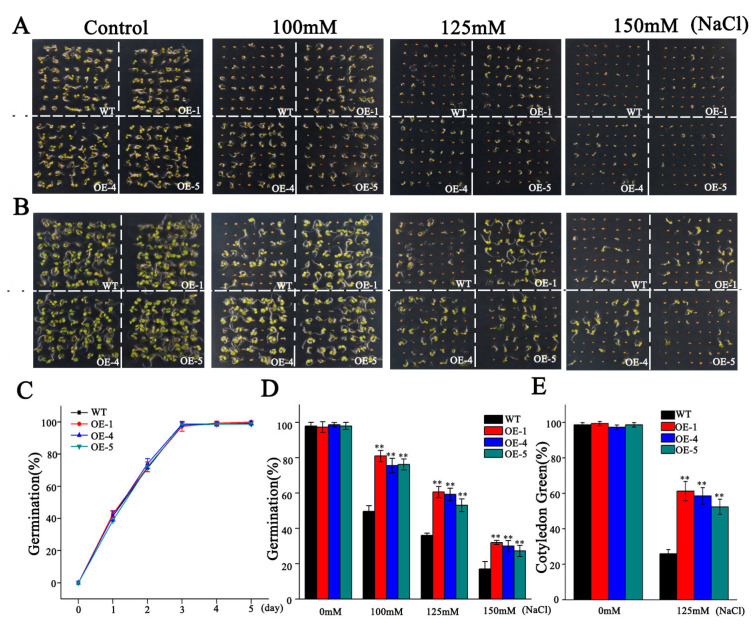
Phenotypic assessment of seed germination and cotyledon greening in WT and transgenic Arabidopsis under NaCl stress. (**A**,**B**) Phenotype of seed germination and cotyledon greening in WT and OE lines exposed to varying NaCl concentrations (0 mM, 100 mM, 125 mM, 150 mM) at designated time points. (**C**) Time-course analysis of germination percentage under control conditions. (**D**) Germination percentages under different NaCl treatments. (**E**) Cotyledon greening percentages under 0 mM and 125 mM NaCl conditions. Statistical significance is indicated by ** *p* < 0.01.

**Figure 7:**
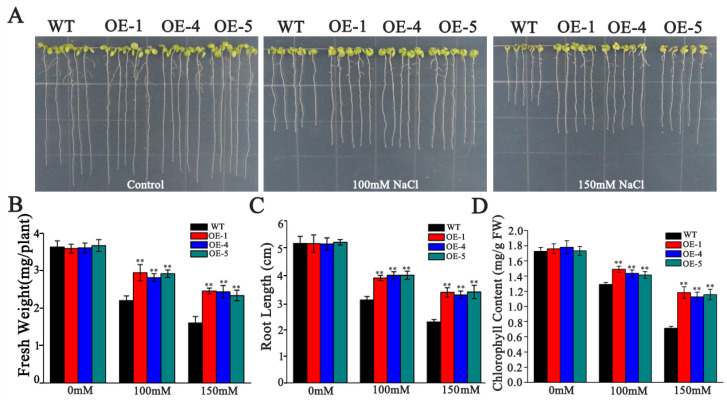
Effects of NaCl stress on plant growth parameters, root length, and chlorophyll content. (**A**) Phenotypic comparison between WT and OE lines under various NaCl concentrations. (**B**) Fresh weight measurements. (**C**) Root length quantification. (**D**) Chlorophyll content analysis. Statistical significance denoted by ** *p* < 0.01.

For the germination assay, seeds from WT and the three transgenic lines were sown on a solid half-strength Murashige and Skoog (1/2MS) medium supplemented with varying concentrations of NaCl (0, 100, 125, or 150 mM). Under control conditions, no significant differences in germination rates were observed between WT and overexpression lines (Figure 6A,C). However, increasing salt concentrations inhibited germination across all genotypes, with the WT exhibiting a significantly greater degree of inhibition compared to the transgenic lines (Figure 6A,D). Furthermore, the proportion of seedlings exhibiting green cotyledons was markedly higher in the overexpression lines relative to WT after five days of germination (Figure 6B,E). These findings suggest that *SlMYB306-like* overexpression enhances salt tolerance during the seed germination phase. Consistent with these observations, there were no significant differences between transgenic and WT plants in root length, fresh weight, or chlorophyll content under non-stress conditions. Conversely, under salt stress, the overexpressing lines demonstrated significantly greater root length, fresh biomass, and chlorophyll content compared to WT (Figure 7).

**Figure 8:**
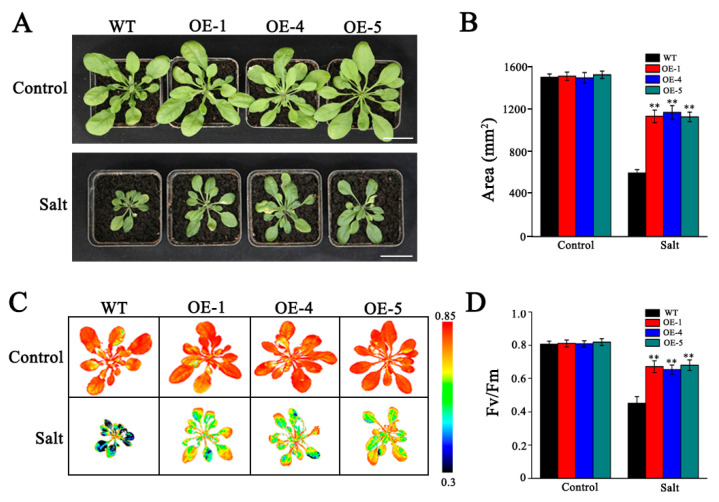
Enhancement of salt tolerance in transgenic Arabidopsis overexpressing SlMYB306-like. (**A**) Phenotypic comparison of WT and OE lines under control and salt stress conditions; scale bars represent 3.5 cm. (**B**) Quantitative analysis of rosette leaf area. (**C**) Chlorophyll fluorescence imaging. (**D**) Quantification of maximum quantum efficiency of photosystem II (Fv/Fm). Statistical significance assessed by ** *p* < 0.01.

To assess whether the enhanced salt tolerance conferred by *SlMYB306-like* overexpression extends to mature plants, three-week-old transgenic and WT plants were treated with 200 mM NaCl for ten days. As illustrated in Figure 8A,C, transgenic plants exhibited markedly reduced salt-induced damage relative to WT, as evidenced by improved growth phenotypes and chlorophyll fluorescence imaging. Quantitative measurements revealed that both the leaf area and Fv/Fm were significantly higher in *SlMYB306-like* overexpressing plants under salt stress compared to WT controls (Figure 8B,D). Collectively, these results demonstrate that the overexpression of *SlMYB306-like* significantly enhances salt tolerance in transgenic *Arabidopsis* at both seedling and mature stages.

### 3.6. Overexpression of SlMYB306-like Attenuates Reactive Oxygen Species Accumulation Under Salt Stress

**Figure 9:**
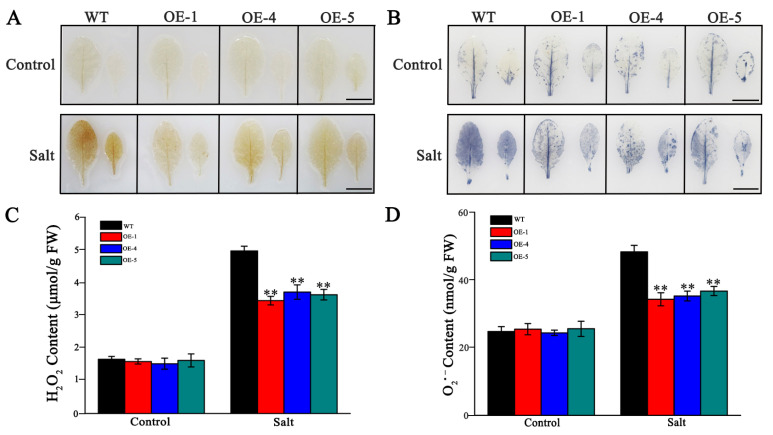
Reactive oxygen species (ROS) analysis in WT and transgenic lines under salt stress. (**A**) DAB staining for hydrogen peroxide (H_2_O_2_). (**B**) NBT staining for superoxide anion (O_2_^•−^). (**C**) Quantitative measurement of H_2_O_2_ content. (**D**) Quantitative measurement of O_2_^•−^ content. Scale bars correspond to 1 cm. Statistical significance indicated by ** *p* < 0.01.

**Figure 10:**
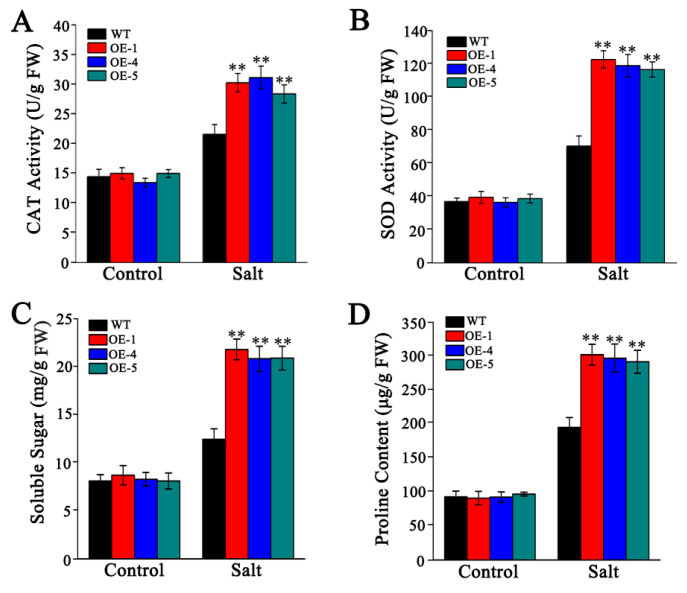
Impact of salt stress on antioxidant enzyme activities and osmolyte accumulation in WT and transgenic plants. (**A**) CAT activity. (**B**) SOD activity. (**C**) Soluble sugar content. (**D**) Proline content. Statistical significance denoted by ** *p* < 0.01.

Exposure to abiotic stress often results in the excessive accumulation of ROS, such as H_2_O_2_ and O_2_^•−^, which can cause oxidative damage to cellular biomolecules. The accumulation of H_2_O_2_ and O_2_^•−^ was assessed histochemically using DAB and NBT staining, respectively. Under control conditions, leaves from both WT and transgenic plants exhibited minimal staining with DAB (indicated by yellowish-brown precipitate) and NBT (indicated by deep blue precipitate). However, under salt stress, WT leaves showed more extensive and intense staining compared to the overexpression lines (Figure 9A,B). Correspondingly, no significant differences in O_2_^•−^ and H_2_O_2_ production rates were observed among the genotypes under normal conditions. In contrast, NaCl treatment significantly elevated the production of these ROS in *Arabidopsis*. Notably, transgenic lines overexpressing *SlMYB306-like* accumulated lower levels of H_2_O_2_ and O_2_^•−^ under salt stress compared to WT (Figure 9C,D). Antioxidant enzymes, including superoxide dismutase (SOD) and catalase (CAT), play critical roles in ROS scavenging. Under salt stress, activities of SOD and CAT were enhanced in the overexpression lines, with their enzymatic activities being approximately 1.8-fold and 1.5-fold higher than those of the WT plants, respectively (Figure 10A,B), consistent with the observed reduction in ROS accumulation.

Furthermore, the transgenic lines demonstrated elevated levels of soluble sugars and proline relative to WT plants under salt stress conditions (Figure 10C,D). Collectively, these findings indicate that the overexpression of *SlMYB306-like* facilitates plant adaptation to saline environments by promoting rapid ROS detoxification and enhancing stress tolerance mechanisms.

### 3.7. Overexpression of SlMYB306-like Mitigates Cellular Damage Induced by Salt Stress

**Figure 11:**
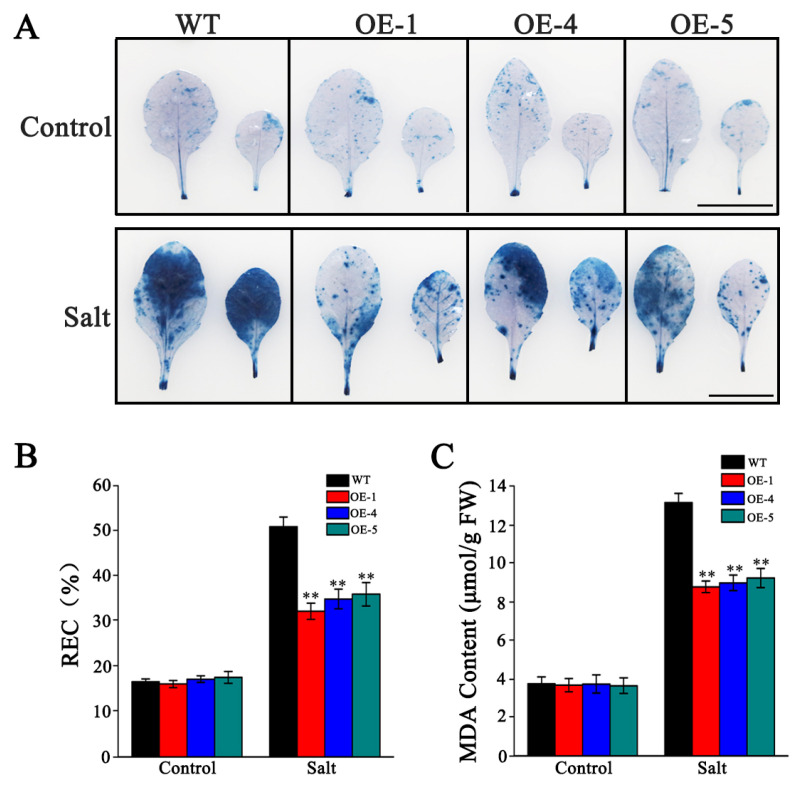
Assessment of cellular damage in WT and transgenic lines. (**A**) Trypan blue staining illustrating cell viability. Upper panels depict plants under normal condition and lower panels show plants under salt stress for 5 days. (**B**) MDA content. (**C**) REC. Scale bars represent 1 cm. Statistical significance indicated by ** *p* < 0.01.

Abiotic stress commonly disrupts cell membrane permeability, leading to cellular injury and potentially cell death. Trypan blue staining, a rapid and reliable assay for detecting cell death, revealed minimal staining in leaves of both WT and transgenic plants under normal conditions. However, under salt stress, WT leaves exhibited more intense staining compared to those of the transgenic lines (Figure 11A). MDA content, an established marker of lipid peroxidation and membrane damage, and REC, an indicator of membrane integrity, were measured to evaluate stress-induced cellular damage [27]. Although salt stress increased MDA levels and REC in all plants, these parameters were obviously lower in the OE lines compared to WT plants (Figure 11B,C). The reduced MDA accumulation and REC in the transgenic plants suggest diminished membrane lipid peroxidation and enhanced plasma membrane stability, thereby indicating that *SlMYB306-like* overexpression confers protection against salt-induced cellular damage.

### 3.8. Overexpression of SlMYB306-like Modulates the Expression of Stress-Responsive Genes

**Figure 12:**
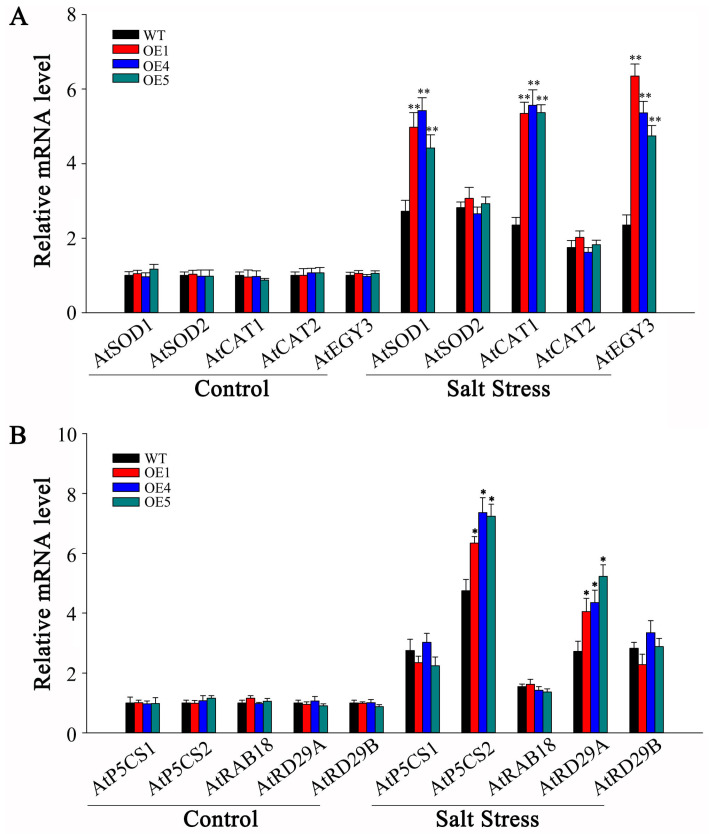
Expression analysis of stress-responsive genes under salt stress conditions. (**A**) Genes involved in antioxidant enzyme biosynthesis and regulation of antioxidant enzyme activity. (**B**) Additional stress-related gene expression profiles. Statistical significance is indicated by * *p* < 0.05 and ** *p* < 0.01.

In order to investigate the molecular mechanisms and physiological roles of the *SlMYB306-like* gene under salt stress conditions, the expression levels of various stress-responsive genes were systematically analyzed. Considering that transgenic plants demonstrate elevated antioxidant enzyme activities and increased accumulation of osmolytes under salt stress, qPCR was employed to assess the transcriptional levels of genes involved in antioxidant enzyme biosynthesis (*AtSOD1*, *AtSOD2*, *AtCAT1*, and *AtCAT2*) as well as genes regulating antioxidant enzyme activity (*AtEGY3*) (Figure 12A). Under salt stress treatment, these genes exhibited differential upregulation across plant lines while, notably, transgenic plants showed a significantly higher expression of *AtSOD1*, *AtCAT1*, and *AtEGY3* compared to WT controls. Subsequently, the expression of genes associated with proline biosynthesis (*AtP5CS1* and *AtP5CS2*) and key components of the ABA signaling pathway (*AtRAB18*, *AtRD29A*, and *AtRD29B*) was examined (Figure 12B). Following salt stress exposure, transgenic lines displayed a significantly elevated expression of *AtP5CS2* and *AtRD29A* relative to WT plants. Collectively, these findings suggest that the enhanced salt tolerance observed in *Arabidopsis thaliana* overexpressing *SlMYB306-like* is likely mediated through the upregulation of *AtSOD1*, *AtCAT1*, *AtEGY3*, *AtP5CS2*, and *AtRD29A*.

## 4. Discussion

The tomato is recognized as one of the most economically important vegetable crops globally. Nevertheless, throughout their growth and development, tomato plants are highly vulnerable to abiotic stresses that significantly compromise both yield and quality, thereby impacting economic outcomes. To adapt to such adverse conditions, plants, including tomatoes, have evolved complex defense mechanisms at morphological, physiological, molecular, and biochemical levels [28]. In particular, identifying genes that regulate salt stress resistance in tomato and elucidating their molecular regulatory pathways can lead to the development of valuable genetic resources. These resources are critical for the development of novel tomato cultivars exhibiting enhanced yield, superior quality, and strong stress resistance, facilitated by advanced techniques such as gene editing and molecular marker-assisted breeding.

Transcription factors (TFs), which function as regulators of gene expression, are known to play pivotal roles in various biological processes. Among them, the MYB family, one of the largest plant-specific TF families, has been implicated in diverse functions including plant growth, development, defense regulation, and stress response. Notably, R2R3-MYB transcription factors have been a focal point of research. However, the functional role of SlMYB306-like in plants remains inadequately characterized. In this study, we investigated the role of SlMYB306-like in *Arabidopsis* under salt stress conditions, aiming to elucidate its comprehensive regulatory mechanisms. Our results demonstrate that SlMYB306-like significantly enhances plant tolerance to salt stress, thereby improving resistance to such environmental challenges.

The MYB transcription factor SlMYB306-like was isolated from tomato, followed by bioinformatics analyses. Using the SMART online tool, it was predicted that the SlMYB306-like protein contains two characteristic SANT-MYB domains (Figure 2), consistent with previous findings in *Gossypium barbadense* [29] and *Macadamia integrifolia* [30]. Amino acid sequence alignment revealed that SlMYB306-like possesses both R2 and R3 MYB domains (Figure 1), indicating its classification within the R2R3-MYB subgroup. While the MYB domain is highly conserved, other regions of the R2R3-MYB proteins exhibit considerable variability [31]. Comparative analysis between SlMYB306-like and MYB proteins from other species showed high similarity within conserved domains, with the closest homology observed with the potato StMYB306-like protein; however, non-conserved regions displayed marked divergence (Figure 1). This pattern is characteristic of transcription factors. Previous studies have established that the functional domains of plant transcription factors are predominantly localized in the nucleus, as exemplified by VhMYB60 and MbMYBC1 [32,33,34]. Consistent with these observations, subcellular localization assays confirmed that SlMYB306-like is a nuclear protein, aligning with the localization patterns of other known MYB transcription factors (Figure 3).

Previous research has established that gene expression in plants exhibits tissue specificity [35]. Both GUS staining and qPCR analyses revealed that the relative expression levels of *SlMYB306-like* in tomato are tissue-specific, predominantly manifesting in the leaves, where it contributes to enhanced stress resistance (Figure 4). Furthermore, *SlMYB306-like* expression was notably induced by NaCl stress (Figure 5), suggesting a potential pivotal role for this gene in modulating plant responses to salt stress. Subsequent investigations demonstrated that the overexpression of *SlMYB306-like* in transgenic *Arabidopsis* conferred increased salt tolerance, as evidenced by phenotypic, physiological, and biochemical assessments (Figure 6 and Figure 7). This study thus provides the first evidence implicating SlMYB306-like as a critical factor in plant salt tolerance. Consistent with these findings, prior studies have indicated that MYB TFs, among the largest TF families, are integral to plant salt stress responses [36,37,38].

Salt stress has been reported to inhibit chlorophyll synthesis or accelerate its degradation, thereby altering chlorophyll content in plant leaves [39,40]. In the present study, salt stress reduced chlorophyll content in *Arabidopsis* leaves, with a more pronounced decrease observed in WT plants compared to those overexpressing *SlMYB306-like* (Figure 7). Impaired synthesis or the accelerated degradation of chlorophyll in plants can affect their normal photosynthesis [41]. Chlorophyll fluorescence analysis serves as a valuable tool for assessing the absorption and utilization of light energy in the process of photosynthesis [42,43], with the Fv/Fm ratio representing a key indicator of photochemical efficiency in photosystem II (PSII) [22]. Compared with normal growth conditions, salt stress significantly diminished Fv/Fm values. Nevertheless, Fv/Fm levels were significantly higher in *SlMYB306-like* overexpression lines compared to WT under salt stress (Figure 8). This indicates that the overexpression of the *SlMYB306-like* gene can effectively alleviate photoinhibition in transgenic *Arabidopsis* under salt stress.

The inhibition of photosynthesis leads to an accumulation of excess electrons and energy within the photosynthetic electron transport chain, triggering a burst of ROS and subsequent peroxidative damage [44]. ROS function as critical signaling molecules in plant defense mechanisms; however, their overproduction can cause detrimental effects on biological macromolecules such as nucleic acids, proteins, and lipids, ultimately leading to cellular damage and death [45]. Our findings demonstrate that *SlMYB306-like* overexpression lines accumulated lower levels of ROS (specifically O_2_^•−^ and H_2_O_2_) induced by salt stress compared to WT plants (Figure 9). Plants have evolved sophisticated ROS detoxification mechanisms to preserve intracellular ROS homeostasis, comprising antioxidant enzymes such as SOD and CAT, as well as antioxidant compounds like proline [46,47]. In this study, *SlMYB306-like* overexpression plants were found to exhibit higher activities of SOD and CAT, as well as a higher proline content (Figure 10). Additionally, ROS can induce lipid peroxidation, causing severe damage to cell membranes. After salt stress treatment, the degree of membrane damage in *SlMYB306-like* overexpression lines was significantly lower than that in WT plants (Figure 11). These findings strongly demonstrate that the overexpression of *SlMYB306-like* can effectively protect plants from ROS-induced cellular damage under salt stress. To elucidate the regulatory mechanisms of SlMYB306-like under salt stress, we analyzed the expression of genes associated with antioxidant enzyme synthesis and activity following salt treatment. The results indicated a significant upregulation of *AtSOD1* and *AtCAT1* transcripts in overexpressing lines, whereas *AtSOD2* and *AtCAT2* expression remained unchanged (Figure 12A). Notably, *AtEGY3* expression was markedly increased in the transgenic plants post salt stress. Given that previous studies have implicated AtEGY3 in enhancing SOD enzyme activity under salt stress conditions [48], it is inferred that the augmented antioxidant enzyme activity observed in *SlMYB306-like* overexpressing plants is mediated both by the upregulation of *AtSOD1* and *AtCAT1* and by the induction of *AtEGY3*, which sustains SOD activity. Furthermore, MYB transcription factors have been reported to enhance stress resistance via ABA-dependent signaling pathways [37,49]. Among them, AtMYB49 from *Arabidopsis thaliana* and IbMYB48 from sweet potato (*Ipomoea batatas*) are both induced by ABA to express, and to then regulate downstream key genes, ultimately achieving the effect of plant salt tolerance [36,38]; meanwhile, the expression of *SlMYB306-like* in this study is also induced by ABA (Figure 5). Consequently, we examined the expression of key genes within the ABA signaling cascade. Our data revealed significant increases in *AtP5CS2* and *AtRD29A* expression in transgenic lines following salt stress (Figure 12B). Collectively, these findings suggest that *SlMYB306-like* overexpression confers the tolerance of salt stress, primarily mediating through upregulating the expression of *AtSOD1*, *AtCAT1*, *AtEGY3*, *AtP5CS2*, and *AtRD29A*.

In summary, a member of the R2R3-MYB transcription factor family in tomato, designated SlMYB306-like, was isolated and characterized for the first time, with subcellular localization confirming its presence in the nucleus. Subsequent analyses revealed that *SlMYB306-like* exhibits tissue-specific expression patterns and is upregulated in response to salt stress. Functional studies demonstrated that SlMYB306-like modulates the expression of key genes associated with ROS scavenging and salt stress response pathways, including *AtSOD1*, *AtCAT1*, *AtEGY3*, *AtP5CS2*, and *AtRD29A*. This regulation results in decreased ROS accumulation and elevated proline content, thereby enhancing salt tolerance in transgenic plants. The insights gained from this investigation into the role of the tomato *SlMYB306-like* gene provide a valuable framework for improving abiotic stress tolerance. Nevertheless, as the functional validation of *SlMYB306-like* has thus far been limited to heterologous expression in *Arabidopsis*, further research is warranted to elucidate its potential role in conferring abiotic stress tolerance in tomato and other species.

## 5. Conclusions

In conclusion, SlMYB306-like, an R2R3-MYB transcription factor from tomato, was isolated and characterized. SlMYB306-like was nuclear localized and had transcription activation activity. The TF exhibited tissue-specific expression (predominant in leaves) and was upregulated under NaCl stress. Its heterologous overexpression in *Arabidopsis* enhanced salt tolerance, as evidenced by higher chlorophyll content, Fv/Fm ratio, proline levels, and SOD/CAT activities, alongside lower ROS accumulation, MDA content, and REC. Mechanistically, it mediated salt tolerance by upregulating key genes (*AtSOD1*, *AtCAT1*, *AtEGY3*, *AtP5CS2*, *AtRD29A*) involved in ROS scavenging and salt stress responses. These findings collectively identify SlMYB306-like as a critical regulator of salt tolerance, providing a valuable genetic resource for improving abiotic stress resistance in crops. Future studies should validate its function in tomatoes and other species.

## Data Availability

Data are contained within the article or Supplementary Material.

## Supplementary Materials

The following supporting information can be downloaded at: https://www.mdpi.com/article/10.3390/biology14111566/s1, Figure S1: qPCR detection of *SlMYB306-like*-overexpressing plants under natural conditions. Four-week-old WT and T_3_ generation transgenic *Arabidopsis* plants were used to detect the expression level of *SlMYB306-like*; Table S1: The primers used for the qPCR reactions; Table S2: Prediction of subcellular localization of SlMYB306-like.

## Abbreviations

The following abbreviations are used in this manuscript:

ABA	abscisic acid
CTAB	cetyltrimethylammonium bromide
DAB	diaminobenzidine
EGFP	enhanced green fluorescent protein
1/2MS	half-strength Murashige and Skoog
H_2_O_2_	hydrogen peroxide
MDA	malondialdehyde
NBT	nitroblue tetrazolium
ORF	open reading frame
O_2_^•−^	superoxide anion radicals
PSI	photosystem I
PSII	photosystem II
REC	relative electrolyte conductivity
ROS	reactive oxygen species
TFs	transcription factors
