# Supplementary material for: Characterization of a Novel Tomato R2R3-MYB Transcription Factor Gene, SlMYB306-like, Conferring Salt Tolerance in Arabidopsis
Source: Biology (Basel). 2025 Nov 7;14(11):1566. doi: 10.3390/biology14111566 (PMC12650281; doi:10.3390/biology14111566)
Supplement: Supplementary file 1 [file biology-14-01566-s001.zip › biology-3966038-supplementary.pdf]

Supplementary file

Table S1 The primers used for the qPCR reactions.

Gene name	Accession No.	Sequence (5′-3′)
SlMYB306-like	XM_004232172	*SlMYB306-like*-F, ACTAGTATGGGTAGACCACCTTGTTGT<br>*SlMYB306-like*-R, CACGTGCTAAATTAATAAATCGTCAT
SlMYB306-like	XM_004232172	*SlMYB306-like-EGFP*-F, GGATCCATGGGTAGACCACCTTGTTGT<br>*SlMYB306-like-EGFP*-R, TCTAGAAATTAATAAATCGTCATTTTC
EF1-α	LOC544055	*EF1α*-F, GGAACTTGAGAAGGAGCCTAAG<br>*EF1α*-R, CAACACCAACAGCAACAGTCT
AtUbiquitin	AT4G05320	*AtUbiquitin*-F, CGGAAAGCAGTTGGAGGATGG<br>*AtUbiquitin*-R, CGGAGCCTGAGAACAAGATGAAG
SlMYB306-like	XM_004232172	*SlMYB306-like*-QPCR-F, AGCTGCTGGTGAAGATTCCGT<br>*SlMYB306-like*-QPCR-R, AGCACATTGTCTTCTTCTACCT
AtSOD1	AT1G08830	*AtSOD1*-F, ATGCTAATCGACATGCTGGTG<br>*AtSOD1*-R, AGTAGCCAGGCTGAGTTCAT
AtSOD2	AT2G28190	*AtSOD2*-F, CTCCAGAAGATGAGTGCCGT<br>*AtSOD2*-R, ACCCTTTCCGAGGTCATCCT
AtCAT1	AT1G20630	*AtCAT1*-F, TGAAATCCTATAAACTCAATATGCTC<br>*AtCAT1*-R, AACAGGAACTAGTACCCTTCTTTAAGC
AtCAT2	AT4G35090	*AtCAT2*-F, CTGGAAAACGTGAGAGGTGC<br>*AtCAT2*-R, GACTTATCAGCCTGAGACCAGT
AtEGY3	AT1G17870	*AtEGY3-F,* TACAACAGCTTGGCTCGTGA<br>*AtEGY3-R,* ATCTCCAAACCGGCGAACAT
AtP5CS1	AT2G39800	*AtP5CS1*-F, GATACGGATATGGCAAAGCG<br>*AtP5CS1*-R, CCAAGTCCAAATCGGAAACC
AtP5CS2	AT3G55610	*AtP5CS2*-F, GCCTGCACCGTTGAAATTGT<br>*AtP5CS2*-R, CAGCACCAAGTCCGAACCTA
AtRAB18	AT5G66400	*AtRAB18*-F, GTCTTACTGCTGAAGGTTCGTCTG<br>*AtRAB18*-R, ATCCAAGATGCTGCGGTTTAGG
AtRD29A	AT5G52310	*AtRD29A*-F, GTGACGACGAAGTTACCTATCTCC<br>*AtRD29A*-R, TCTCCGCCACATAATCTCTACCC
AtRD29B	AT5G52300	*AtRD29B*-F, GCAAGCAGAAGAACCAATCA<br>*AtRD29B*-R, CTTTGGATGCTCCCTTCTCA
35S		TACGCAGCAGGTCTCTCAAGACGAT

Table S2 Prediction of subcellular localization of SlMYB306-like.

Location weight	LocDB	PotLocDB	Neural Nets	Pentamers	Integral
Nuclear	5.0	5.0	0.00	0.14	8.53
Plasma membrane	0.0	0.0	0.96	0.22	0.87
Extracellular	0.0	0.0	0.96	0.79	0.00
Cytoplasmic	0.0	0.0	0.00	1.55	0.00
Mitochondrial	0.0	0.0	0.00	1.71	0.00
Endoplasm. retic.	0.0	0.0	0.00	0.24	0.00
Peroxisomal	0.0	0.0	0.96	0.00	0.10
Golgi	0.0	0.0	0.11	0.31	0.00
Chloroplast	0.0	0.0	0.00	0.21	0.06
Vacuolar	0.0	0.0	0.00	0.08	0.44

The data have been obtained with ProtComp 9.0 (http://linux1.softberry.com/berry.phtml). Integral prediction of SlMYB306-like protein location: Nuclear with score 8.53.

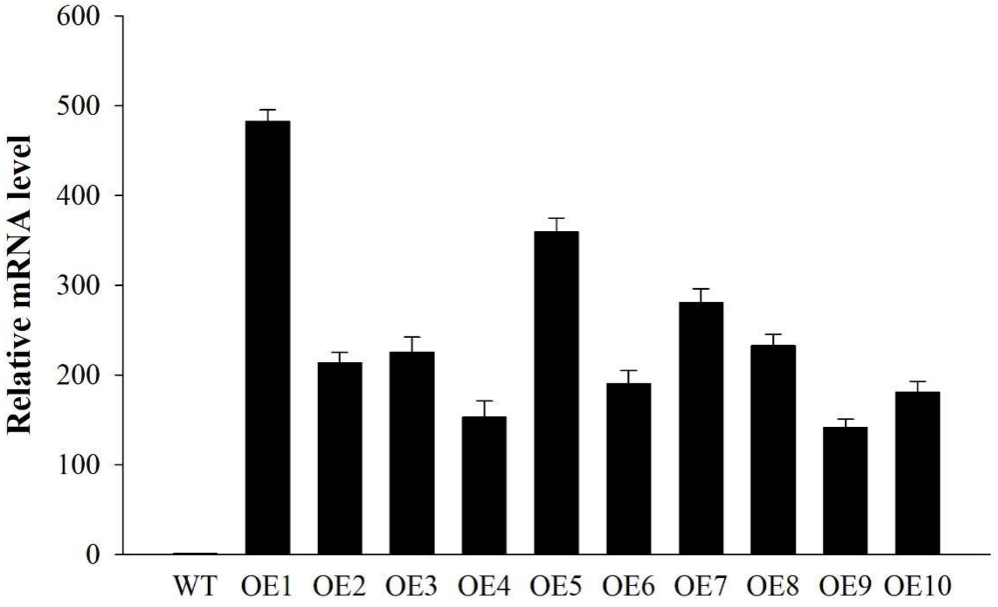

Figure S1 qPCR detection of *SlMYB306-like*-overexpressing plants under natural conditions. Four-week-old WT and T_3 generation transgenic *Arabidopsis* plants were used to detect the expression level of *SlMYB306-like*.
